# Facile Gram-Scale Production of Cu/Cu_2_O Core/Shell Nanoparticles Densely Embedded in a Porous Carbon Framework for Cost-Effective Peroxidase Mimicking

**DOI:** 10.1021/acsami.5c05766

**Published:** 2025-06-19

**Authors:** Yuzhen Cai, Zhanping Xiao, Tianqi Cheng, Bo Yuan, Yifan Cui, Jian Lin Chen, Yufei Zhao, Pi-Tai Chou, Yung-Kang Peng

**Affiliations:** 1 Department of Chemistry, 53025 City University of Hong Kong, Hong Kong 999077, Hong Kong SAR; 2 Department of Chemistry, 33561 National Taiwan University, Taipei 106319, Taiwan; 3 Department of Applied Science, School of Science and Technology, 66386 Hong Kong Metropolitan University, Hong Kong 999077, Hong Kong SAR; 4 State Key Laboratory of Chemical Resource Engineering, 47832 Beijing University of Chemical Technology, Beijing 100029, China

**Keywords:** peroxidase mimetics, gram-scale production, Cu-based nanozymes, cost-effectiveness, biomolecule detection

## Abstract

Natural enzymes are efficient catalysts but face high costs and instability, leading to the development of artificial enzymes like nanozymes. While noble metals commonly demonstrate high peroxidase (POD)-like activity, their expense limits their practical use. In contrast, 3d transition metal oxides, though less active, are more cost-effective due to their natural abundance, with Cu­(I) emerging as a promising candidate. However, maximizing POD-like activity in small-sized Cu_2_O nanoparticles (NPs) often requires complex synthetic processes and labor-intensive purification, making mass production challenging. To address these issues, it is crucial to develop POD nanozymes with simplified production methods that would reduce costs and facilitate their real-world applications. Herein, we present a straightforward and scalable method for preparing Cu/Cu_2_O core/shell NPs densely embedded within a porous carbon-based framework by calcining Cu precursor and polyvinylpyrrolidone (PVP) at elevated temperatures in nitrogen. The resulting samples with Cu/Cu_2_O NPs around 15 nm in size can be obtained at temperatures below 600 °C. Importantly, they can be used directly without purification, significantly reducing production costs compared to natural enzymes. The sample obtained at 300 °C, exhibiting the highest Cu­(I) content, displays optimal POD-like activity and was further demonstrated in the detection of glutathione and glucose. This study is anticipated to guide the future development of scalable and cost-effective POD nanozymes for practical applications.

## Graphical abstract

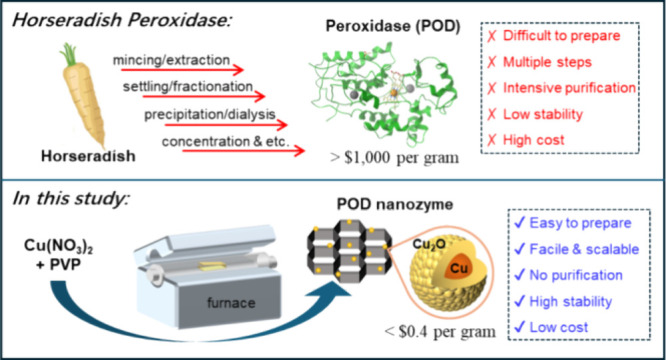

## 1 Introduction

Natural enzymes efficiently catalyze biochemical reactions; however, they encounter intrinsic limitations such as high production costs and poor stability. To overcome these challenges, researchers have developed various enzymatic mimics, commonly referred to as artificial enzymes.^1,2^ Among these, nanozymes are nanosized materials that exhibit enzyme-like catalytic activity.^3–5^ Since the discovery of Fe_3_O_4_ NPs with POD-like activity,^6^ a wide range of materials has been reported to exhibit this property, finding applications across various fields, including biomolecular sensing,^7^ disease treatment,^8^ and environmental protection.^9^ Despite the rapid advancement in this field over the past two decades, several challenges remain to be addressed to facilitate the transition from laboratory research to industrial implementation.

First and foremost, the selection of material elements is crucial in the design of POD nanozymes for practical use as different elements vary in terms of natural abundance and cost. Scheme 1a shows the normalized activity of common POD nanozymes found in the literature ranging from metal oxides, metals to alloys (see detailed calculations in the Section 2 and the obtained parameters in Table S1).^10–24^ Noble metals such as Pt, Pd, Ru, and Ir, along with their alloys, demonstrate exceptional POD-like activity; however, their high production costs hinder the implementation of the applications demonstrated in the literature. While metal oxides, particularly those of the 3d transition elements, display lower POD-like activity (Scheme 1a), their natural abundance renders them relatively cost-effective. The origin of transition metal oxides as POD mimetics has recently been elucidated through the Fenton/Fenton-like mechanism.^19^ As shown in Scheme 1b, the redox reaction between M^*n*+^ and H_2_O_2_ generates OH radicals (step (i)), which then oxidize substrates such as 3,3′,5,5′-tetramethylbenzidine (TMB), causing a color change (step (ii)). These radicals also oxidize H_2_O_2_ to produce HO_2_ radicals (step (iii)) for the regeneration of M^*n*+^ (step (iv)). Since step (i) is the rate-limiting step, a positive correlation has been observed between the reaction rate of M^*n*+^/M^(*n*+1)+^ with H_2_O_2_ in Cu/Fe-based nanozymes (Scheme 1c) and their POD-like activity (Scheme 1a).^19,20^ Among the 3d transition metal cations, Cu­(I) is anticipated to be the most cost-effective material for constructing POD nanozymes.

**Scheme 1:**
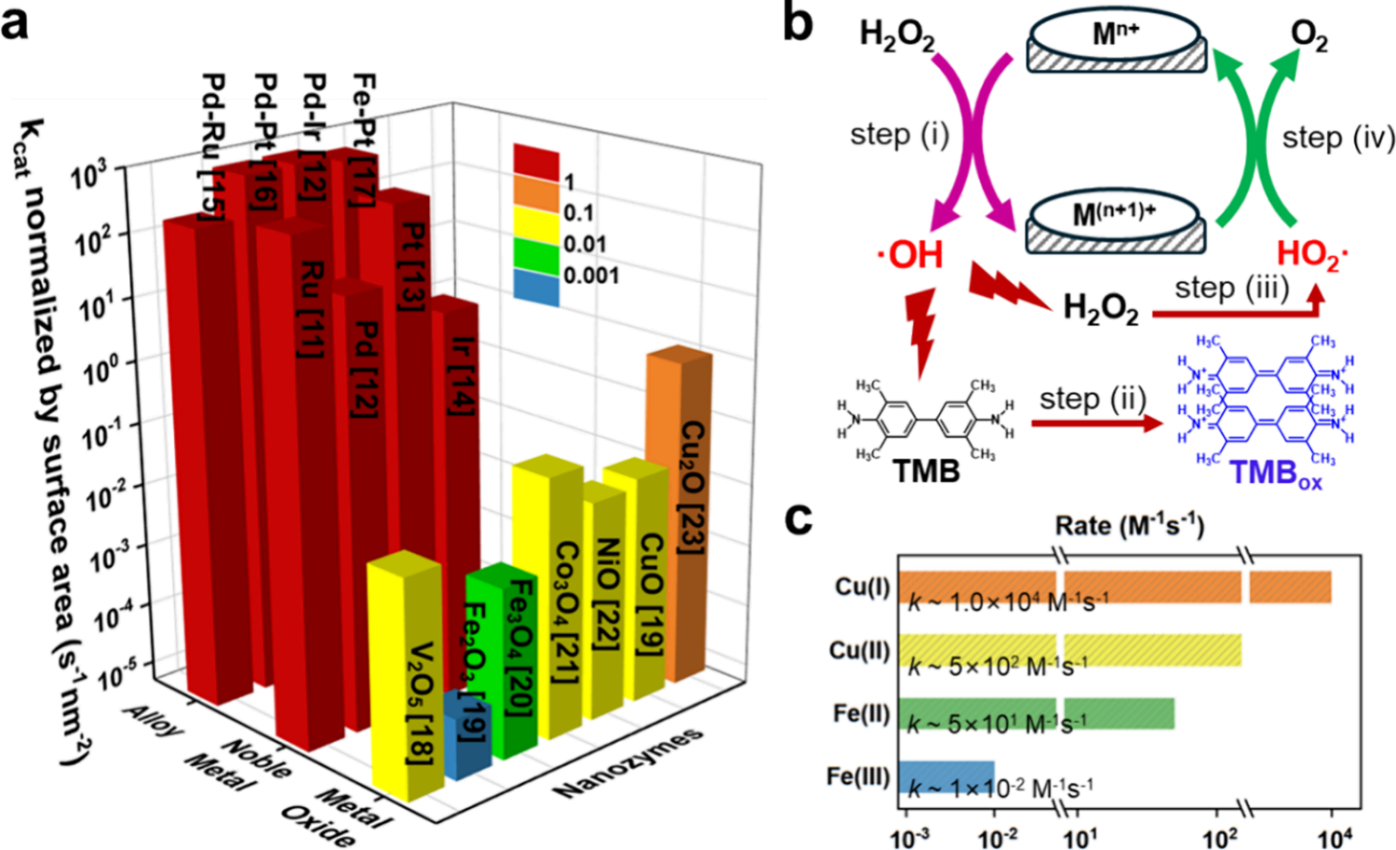
(a) Normalized POD-Like Activity of Various Metal Oxides, Metals, and Alloys Reported in the Literature. (b) Proposed Mechanism for Transition Metal Oxides Acting as POD Mimetics. (c) Reported Reaction Rates of Cu­(I), Cu­(II), Fe­(II), and Fe­(III) with H_2_O_2_

However, the preparation of corresponding oxide, Cu_2_O, often involves multiple steps and labor-intensive purification processes, such as repeated washing and centrifugation.^25–30^ To maximize Cu­(I) exposure, their small-sized synthesis (typically <20 nm) further necessitates precise control over precursor concentration and the incorporation of various surfactants^25–27^ or supports^28–30^ to prevent particle aggregation. These complexities not only hinder mass production but also lead to variations in quality and increased production costs. Cu­(I)-based single-atom nanozymes^31,32^ and metal organic frameworks (MOFs)^33^ require additional characterization to ensure the proper coordination of their active centers, significantly increasing both synthesis costs and time. To overcome these challenges, it is essential to develop POD nanozymes that can be produced using a scalable approach with minimal steps, maintaining activity while eliminating purification needs. Meeting these criteria would not only greatly reduce costs but also facilitate their integration into practical applications.

To meet these criteria, we develop a facile method for synthesizing Cu/Cu_2_O core/shell NPs densely populated in a porous carbon framework. This is achieved by calcining a mixture of a Cu precursor and PVP in nitrogen. The resulting samples can be used directly as POD mimetics without purification, making them suitable for large-scale production. Cu/Cu_2_O NPs approximately 15 nm in size can be achieved for samples obtained at temperatures below 600 °C, with those obtained at 300 °C exhibiting the highest surface Cu­(I) and, consequently, the greatest POD-like activity. These NPs are embedded within a free-standing carbon host, which provides a high particle density per unit volume to enhance the reaction efficiency. The practicality of the optimized sample was further assessed in its ability to detect biologically important molecules, such as glutathione and glucose.

## 2 Materials and Methods

### 2.1 Calculation of the Normalized *k*_cat_ in the Literature

The calculation of the normalized *k*_cat_ is based on a recent report,^20^ with equations shown below:

$$\left[P\right]=\frac{m_{\mathrm{total}}}{\rho \times V_{\mathrm{NPs}}\times N_{A}\times V_{\mathrm{total}}}$$

$$k_{\mathrm{cat}}=V_{\max}/\left[P\right]$$

$$\mathrm{Normalized}k_{\mathrm{cat}}=k_{\mathrm{cat}}/S_{\mathrm{NPs}}$$

where [*P*] is the particle concentration in the reaction solution; *m*_total_ and *V*_total_ represent the total weight of nanozymes used for the reaction and the total volume of the reaction solution, respectively, as reported in the literature; ρ is the material density of the nanozymes (e.g., 6.0 g/cm^3^ for Cu_2_O); *N_A_* is Avogadro constant (i.e., 6.02 × 10^23^); *V*_NPs_, and *S*_NPs_ refer to the particle volume and surface area of the nanozymes, respectively, determined based on their morphology. *V*_max_ is the Michaelis–Menten parameter reported in the literature.

### 2.2 Chemicals

Cupric­(II) nitrate (Cu­(NO_3_)_2_, Sigma-Aldrich); PVP (MW = 1300,000, Sigma-Aldrich); hydrogen peroxide solution (30% (w/w) in H_2_O, Sigma-Aldrich); sodium acetate (Sigma-Aldrich); acetic acid (AcOH, Sigma- Aldrich), TMB (Aladdin); 5,5-dimethyl-1-pyrroline *N*-oxide (DMPO, > 97%, Sigma-Aldrich); glucose (Sigma-Aldrich); glucose oxidase (GO_*x*_, 145.2 kU·g^–1^, Sigma-Aldrich); POD from horseradish (type VI, essentially salt-free, lyophilized powder, ≥150 units/mg solid (using pyrogallol), Sigma-Aldrich); human serum sample (Solarbio.); fetal bovine serum (FBS) (Life technologies); and horse serum sample (Life technologies) were used. Water was purified by a Millipore Milli-Q System (resistivity is 18.2 MΩ·cm).

### 2.3 Synthesis of Cu-*X* samples

The Cu-*X* samples were synthesized by calcinating the aqueous mixture of Cu­(NO_3_)_2_ and PVP with a mass ratio of 1.5:1 in nitrogen. This calcination process took place at elevated temperatures, with a heating rate of 5 °C/min and then maintained for 1 h upon reaching the target temperature (200 to 700 °C). The resulting sample was used directly without any additional purification steps.

### 2.4 Material Characterization

The crystalline phases of Cu-*X* samples were identified by X-ray diffraction (XRD, D2 PHASER second gen, Bruker) with monochromatized Cu Kα radiation. The morphology of Cu-X samples was investigated by a transmission electron microscope (TEM, Philips Technai 12) and a scanning electron microscope (QUATTRO S). The corresponding lattice spacings were identified by a high-resolution TEM (HRTEM, JEOL 1200 F) with an acceleration voltage of 200 kV. X-ray photoelectron spectroscopy (XPS) data were recorded on a Themo Scientific Neesa system (12 kV, 6 mA), with binding energies calibrated using the C_1s_ peak at 284.8 eV. Fourier transform infrared attenuated total reflection (FTIR-ATR, PerkinElmer Spectrum 100) spectroscopy was applied to investigate the chemical bonds of samples in the range of wavenumbers 400–4000 cm^–1^ at a resolution 4 cm^–1^ (number of scans, 32). All of the presented spectra were obtained by subtraction of the background of air.

### 2.5 POD-Like Activity

The POD-like activity was assessed by mixing 150 μL of the Cu-*X* solution (1.0 mg/mL), 150 μL of TMB solution (5 mM), 1.05 mL of acetate buffer (50 mM, pH = 4.0), and 150 μL of H_2_O_2_ solution (5 mM), resulting in final concentrations of 0.5 mM for both TMB and H_2_O_2_. The UV–vis absorption spectra were measured using a Cary 60 UV–vis spectrophotometer (Agilent Technologies) at 2 min intervals for a total of 20 min. All spectra were obtained by subtracting the absorption of the Cu-*X* samples in acetate buffer as a control.

### 2.6 EPR Detection of OH and HO_2_ Radicals

Electron paramagnetic resonance (EPR) measurements were carried out in ambient conditions on an ADANI SpinscanX spectrometer operating at 100 kHz field modulation at room temperature. DMPO was chosen as the spin trap. For the detection of OH radicals, 100 μg of Cu-X samples was dispersed respectively in an as-prepared 1 mL acetate buffer solution (pH 4) containing 50 mM DMPO and allowed to react for 10 min. Subsequently, Cu-*X* samples were removed by filtration, and the supernatant was measured. Methanol was used as the solvent for the detection of HO_2_ radicals.

### 2.7 Temperature and pH Stability Study

The stability study was conducted by incubating Cu-300 and POD (0.0075 U/mL) under various temperatures (from 0 to 55 °C) or pH conditions (from 2 to 13, adjusted using NaOH with acetate, PIPES, or PBS buffer solutions) for 120 min. Following this incubation, their POD-like activity was tested, as described above.

### 2.8 Detection of Glutathione and Glucose

For glutathione (GSH) detection, 150 μg of Cu-300 and Cu-400 were dispersed in 1.5 mL of acetate buffer solution (pH 4) containing 0.5 mM TMB and H_2_O_2_. This mixed solution was allowed to react for 18 min before the removal of NPs by centrifugation at 10,000 rpm for 2 min. The absorbance at 652 nm of the supernatant was recorded and denoted as *A*_0_. Then, freshly prepared GSH solutions were mixed with the solution above (final GSH concentration: 1, 2, 5, 10, 25, 50, 70, and 80 μM) and allowed to react for 10 min. The absorbance at 652 nm was recorded again and denoted as *A*. The change in absorbance at 652 nm (i.e., *A*–*A*_0_) was plotted as a function of the GSH concentration.

For glucose detection, glucose solutions with concentrations ranging from 0.01 to 3 mM (0.01, 0.02, 0.05, 0.075, 0.1, 0.2, 0.5, 1, and 3 mM) were prepared. A 150 μL aliquot of each glucose solution was incubated with 150 μL of glucose oxidase (GO_*x*_, 1 mg/mL) for 30 min at 37 °C to produce H_2_O_2_ (denoted as solution A). Separately, solution B was prepared by mixing 150 μL of TMB solution (5 mM), 1.05 mL of acetate buffer (50 mM, pH = 4.0), and 150 μL of Cu-300 or Cu-400 solution (1 mg/mL). Solution *A* was then combined with solution *B* and incubated for an additional 20 min at 37 °C. The final concentrations of glucose in the reaction mixtures were 1, 2, 5, 7.5, 10, 20, 50, 100, and 300 μM, respectively. The formation of TMB_ox_ was quantified using UV–vis spectroscopy at 652 nm. The limit of detection (LOD) for GSH and glucose was calculated using the following formula:

$$\mathrm{LOD}=\frac{3\times \mathrm{SD}}{B}$$

where *B* represents the slope of the calibration curve, and SD is the standard deviation of the *y*-intercept. This approach ensured accurate and reproducible quantification of GSH and glucose concentrations based on the POD-like activity of Cu-300 and Cu-400. For the selectivity test, solutions containing agarose, D-sorbitol, glucose, sodium chloride, cholesterol, l-arginine, glutamic acid, l-cysteine, and ascorbic acid (1 mM) were further tested.

### 2.9 GSH and Glucose Detection in FBS

The FBS sample (purchased from Solarbio, Beijing, China) was first treated by centrifugation at 10,000 rpm for 10 min, and the supernatant was collected and diluted 10 times. For GSH detection, spiked samples were prepared by adding a GSH standard solution (final concentration: 10, 20, and 30 μM). GSH levels were determined using the calibration curve after measuring absorbance of TMB_ox_ at 652 nm. For glucose detection, each serum sample was diluted 10 times by PBS buffer before use. 150 μL of the serum sample was mixed with 50 μL of GO_*x*_ (10 mg/mL) and incubated at 37 °C for 30 min. This solution was then mixed with solution *B* prepared above and allowed to react at 37 °C for another 20 min. UV–vis was used to record the generation of the corresponding TMB_ox_ at 652 nm. Parallel experiments were conducted three times, and the data were presented as mean ± SD. The relative standard deviation (RSD) was obtained by dividing the standard deviation by the corresponding average value. Recovery value was calculated by dividing the averaged concentration obtained by the corresponding known concentration.

## 3 Results and Discussion

### 3.1 Preparation and Characterization

The title samples can be readily prepared by calcining an aqueous mixture of Cu­(NO_3_)_2_ and PVP (referred to as Cu-PVP) under nitrogen at elevated temperatures (Figure 1a). The resulting products, denoted as Cu-*X* (where *X* represents the calcination temperature), were used directly without purification. Gram-scale production was successfully demonstrated using a laboratory furnace (Figure S1). Their scanning electron microscopy (SEM) images in Figure 1b reveal porous frameworks in samples obtained below 600 °C, while they collapse at temperatures exceeding 700 °C. The corresponding elemental mapping shows a uniform distribution of C, N, O, and Cu signals in each sample (Figure S2). TEM images reveal the presence of NPs densely dispersed within the framework (Figure 1b), with sizes around 15 nm at temperatures below 600 °C (Figure 1c; see the statistics in Figure S3). However, the size of NPs increases sharply to approximately 90 nm for Cu-700, likely due to the collapse of the framework. High-resolution TEM (HRTEM) analysis suggests that these NPs exhibit a Cu/Cu_2_O core/shell structure, with lattice spacings of ca. 0.21 nm/0.24 nm (Figure 1d; see Figure S4 for other samples), corresponding to their (111) planes.^34^ This finding is supported by the XRD patterns (Figure 1e), which reveal a dominant Cu signal that increases with the calcination temperature and a weak Cu_2_O signal. In contrast, these diffraction signals were absent in the Cu-PVP mixture. The crystallite sizes of Cu and Cu_2_O in the Cu-*X* samples were further approached from the full width at half-maximum (fwhm) of the corresponding XRD peaks (Table S2), with Cu ranging from 7 to 11 nm and Cu_2_O from 1.0 to 1.8 nm. In conjunction with the HRTEM images of these samples, the weak XRD signal of Cu_2_O can be primarily attributed to peak broadening effects resulting from its small crystallite size (cf. Cu). As highlighted for Cu-300 in Figure 1d, we conclude that the shell is polycrystalline, consisting of numerous tiny Cu_2_O crystallites, while the core is single-crystalline Cu of a larger size. Overall, our findings indicate that samples obtained at temperatures below 600 °C can produce Cu/Cu_2_O core/shell NPs as small as ca. 15 nm densely embedded within a porous framework. Notably, using Cu­(NO_3_)_2_ alone yields only CuO with sizes over 500 nm (Figure S5), highlighting the crucial role of PVP in reducing Cu­(II) and preventing particle sintering during synthesis.

**Figure 1:**
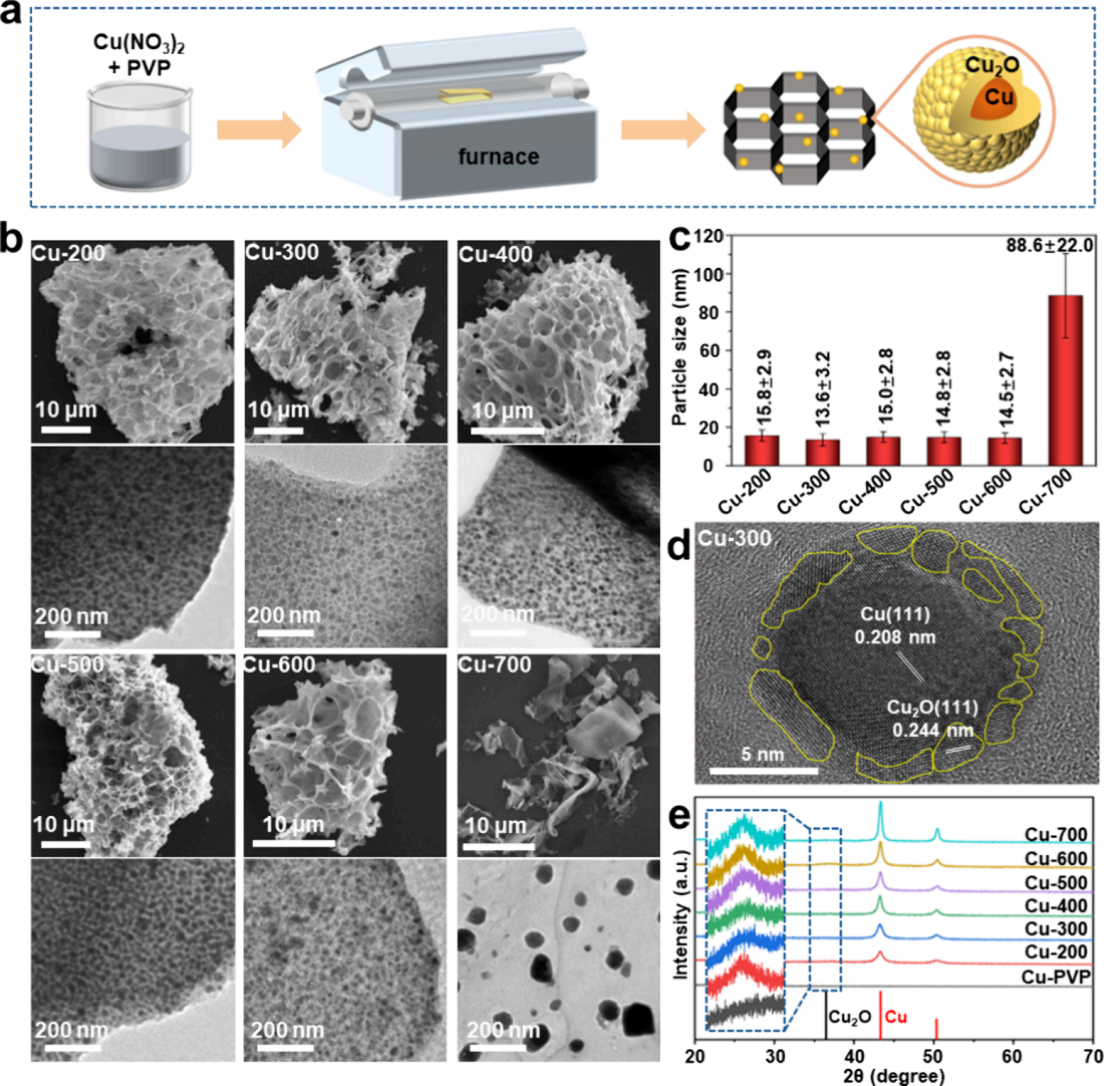
(a) Schematic illustration of the synthesis of Cu/Cu_2_O core/shell NPs embedded in a porous framework. (b) SEM and TEM images of the samples prepared at elevated temperatures. (c) Size distribution of NPs in the samples and (d) corresponding HRTEM image. (e) XRD patterns of the samples.

XPS was used to study the chemical states of C, N, O, and Cu in samples before and after calcination at temperatures below 600 °C. The XPS C_1s_ spectra were similar across all samples (Figure S6), while the other elements were found in different forms, especially after calcination. For example, the N_1s_ signals of nitrate-N (from the Cu precursor) and pyrrolic-N (from PVP) in the Cu-PVP mixture disappeared (Figure 2a), giving rise to graphitic and pyridinic N in the calcined samples, which are commonly observed for N-doped carbon materials (or supports).^35,36^ The XPS O_1s_ spectra reveal a similar trend (Figure 2b), with the nitrate-O signal in the Cu-PVP mixture shifting from 532.5 eV to surface oxygen species at 531.6 eV in the calcined samples.^37^ Notably, despite the presence of a weak XRD peak associated with Cu_2_O in the calcined samples, no signal for lattice-coordination-saturated oxygen around 529.8 eV was detected. This suggests that the majority of the oxygen atoms in the Cu_2_O shell are coordination unsaturated, a result of its polycrystalline nature. In the Cu_2p_ region (Figure 2c), a dominant Cu­(II) signal appears at 935.2 eV for the Cu-PVP mixture. This species was reduced to Cu­(I) and Cu(0) in the calcined samples, likely due to the nitrogen in PVP, as indicated by the emergence of signals at 933.8 and 932.6 eV.^38^ Both Cu­(I) and Cu(0) signals observed for the Cu-X samples can be attributed to the formation of the Cu/Cu_2_O core/shell NPs. The absence of identifiable lattices (from HRTEM) and diffraction peaks (from XRD) for Cu­(II)O in these calcined samples further suggests that their Cu­(II) signal in XPS should originate from residual Cu­(II) in the carbon-based framework. Notably, peak deconvolution analysis reveals that the intensity of the Cu­(I) peak (red highlighted, Figure 2c) increases with calcination temperature up to 300 °C, followed by a significant decrease at 400 °C, and then a gradual rise again at higher temperatures. A similar trend is observed in the Cu­(I)/Cu­(II) ratios calculated from their deconvoluted peak areas (Figure S7), yielding values of 0.06 for Cu-PVP, 0.56 for Cu-200, and 1.44 for Cu-300 (Figure 2d). This ratio sharply decreased to 0.41 for Cu-400 but gradually increased again to 0.67 for Cu-500 and 1.22 for Cu-600. The change in Cu­(I) intensity and Cu­(I)/Cu­(II) ratios with the calcination temperature is still unclear. However, it is unlikely to be related to different functional groups produced at elevated temperatures, as all calcined samples exhibit similar infrared spectra (Figure S8). Since Cu/Cu_2_O NPs readily form at 200 °C, when PVP begins to decompose (Figure 2e), the variation in Cu­(I)/Cu­(II) ratios at higher temperatures should be closely associated with the dynamic interplay between Cu­(I) in the surface polycrystalline Cu_2_O layer and residue Cu­(II) in the carbon-based framework during PVP decomposition.

**Figure 2:**
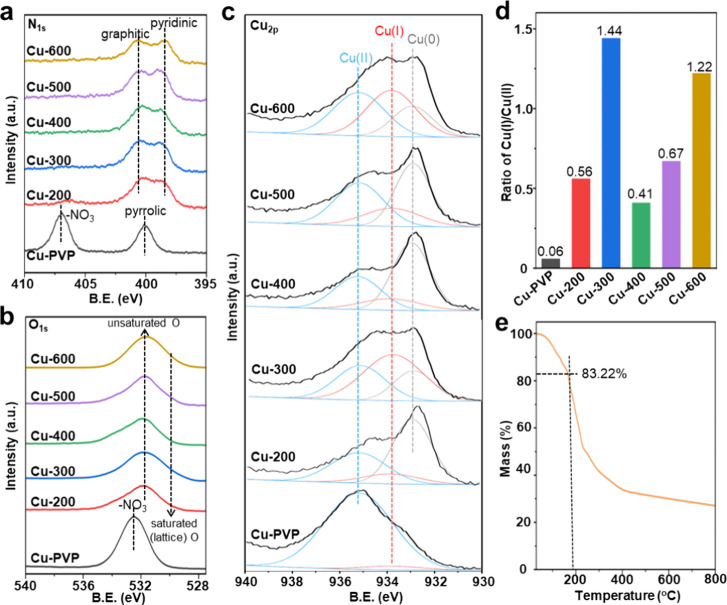
XPS analysis of the samples in the (a) N_1s_, (b) O_1s_, and (c) Cu_2p_ regions. (d) Calculated Cu­(I)/Cu­(II) ratios for the samples. (e) Decomposition of theCu-PVP mixture at elevated temperatures under nitrogen.

### 3.2 Catalytic Assessment

The POD-like activity of the samples was evaluated through the catalytic oxidation of TMB with H_2_O_2_ to form TMB_ox_ (Scheme 1b), which results in a color change from colorless to blue (λ = 652 nm). Figure 3a shows their time-dependent production of TMB_ox_ monitored by UV–vis spectroscopy in 20 min. Notably, the Cu-PVP mixture and Cu-200 exhibited comparable performances. Since the Cu-PVP mixture is fully dissolved in the aqueous solution, all of its Cu­(II) can participate in the reaction. In contrast, Cu-200 has only a small amount of Cu­(I) in the surface of Cu/Cu_2_O NPs and residual Cu­(II) in the carbon-based framework available for the reaction. This implies that Cu­(I) is a more active species than Cu­(II) in a POD-like activity, as suggested by its reaction rate with H_2_O_2_ in Scheme 1c. By comparing the activities among the Cu-X samples, we observed a catalytic trend that aligns with the variation in their Cu­(I)/Cu­(II) ratios. Specifically, the activity increases from Cu-200 to Cu-300, then significantly decreases for Cu-400, followed by a gradual rise for Cu-500 and Cu-600. This relationship is also evident in the initial rate constants calculated from Figure 3b and correlated in Figure 3c. These findings collectively indicate that Cu­(I) from the surface polycrystalline Cu_2_O layer is the key contributor to the observed POD-like activity, while the contribution from residual Cu­(II) in the carbon-based framework is nearly negligible. To further confirm that the POD-like activity of the Cu-X samples is mediated by OH and HO_2_ radicals (Scheme 1b), we employed EPR with DMPO as a radical trap. As expected, DMPO–OH, exhibiting a 1:2:2:1 resonance intensity (Figure 3d), and DMPO-OOH, displaying six resonance lines (Figure 3e), were both observed across all samples, with their overall intensities reflecting the same trends as their POD-like activity. Since all of the Cu-*X* samples have similar configurations (i.e., similarly sized Cu/Cu_2_O NPs dispersed within a carbon-based framework), their observed POD-like activity is thus primarily determined by the amount of Cu­(I) present in their surface polycrystalline Cu_2_O layer.

**Figure 3:**
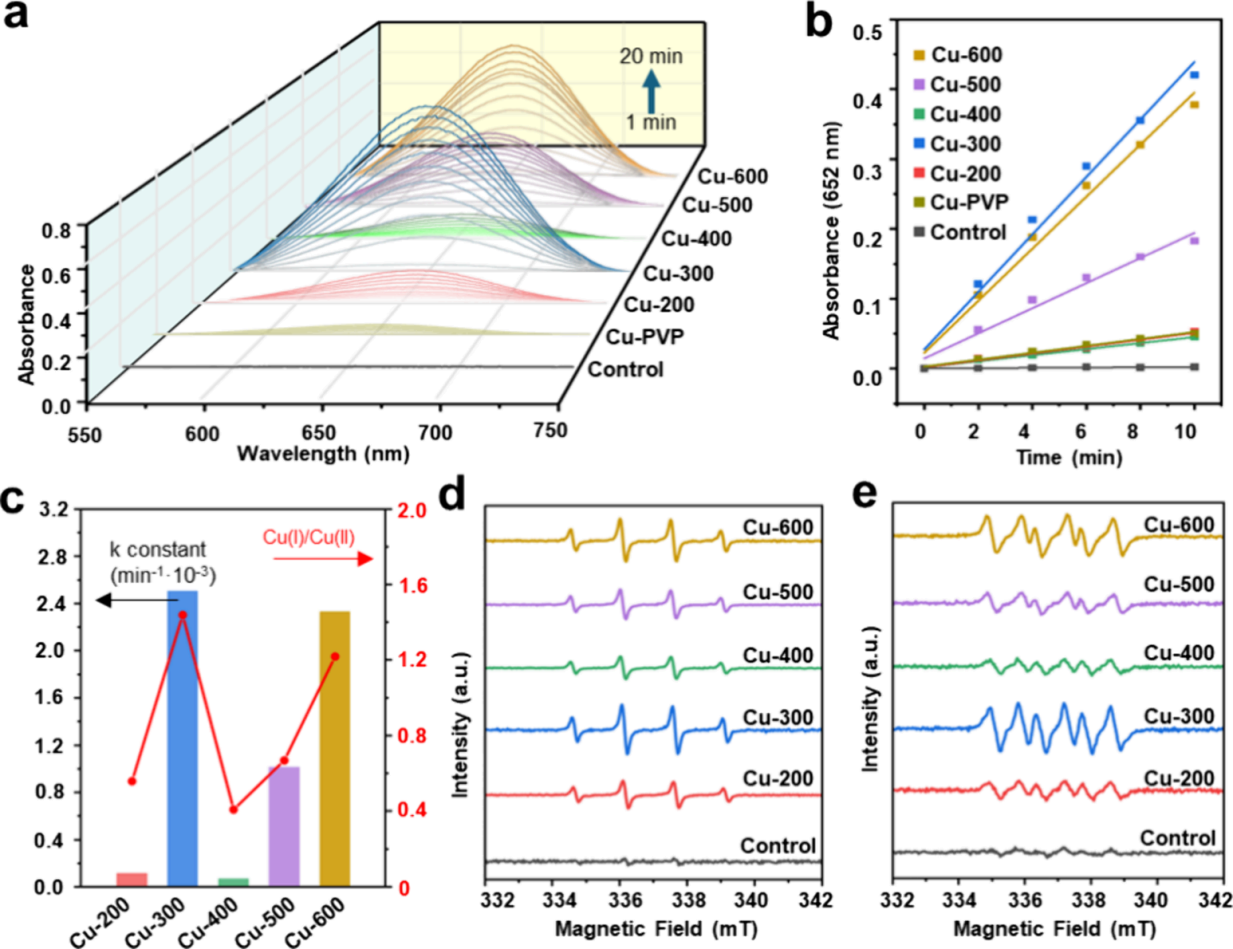
(a) Time-dependent UV–vis spectra monitoring the reaction across the samples. (b) Initial rate analysis for Cu-X samples and (c) correlation with their Cu­(I)/Cu­(II) ratios. DMPO-assisted EPR monitoring of the generation of (d) OH and (e) HO_2_ radicals over Cu-X samples in the presence of H_2_O_2_.

### 3.3 Cost-Effectiveness, Stability, and Recyclability

The yield of the Cu-*X* samples in each batch was assessed based on the quantities of raw materials used and their conversion efficiencies to products (Table S3), allowing us to estimate the production cost per gram (Table S4). As summarized in Figure 4a, their estimated production cost ranges from $0.16 to $0.36 per gram. These costs are significantly lower than those of POD enzymes (∼$1000 per gram), which require complex extraction and purification processes (Figure S9).^39^ Given that Cu-*X* samples exhibit varying activities per mass, the cost-effectiveness of each sample was further calculated by normalizing their initial rate constants against the production cost. As also displayed in Figure 4a, Cu-300 demonstrated the best cost-effectiveness compared to the other samples and, most importantly, POD enzymes, indicating superior economic viability for large-scale production. To evaluate the consistency of Cu-300 across batches and the effect of purification, we prepared three separate batches to compare their POD-like activity, both with and without washing (Figure 4b). The purification procedure involved dispersing the products in deionized water, sonicating for 10 min, centrifuging at 14,000 rpm for 10 min, and then drying them in a 60 °C oven overnight. Notably, the different batches exhibited minimal changes before and after washing, indicating that product quality was consistent across batches and that purification had a negligible effect on the POD-like activity. The concentration of Cu in the washing supernatant was further analyzed by inductively coupled plasma mass spectrometer (ICP-MS) (Table S5), revealing minor leaching of Cu (<2%). Since the POD-like activity remained unaffected after purification, the leached Cu likely originated from the significantly less active Cu­(II) ions in the carbon framework. These findings demonstrate that the Cu-*X* samples can be used directly without the need for purification.

**Figure 4:**
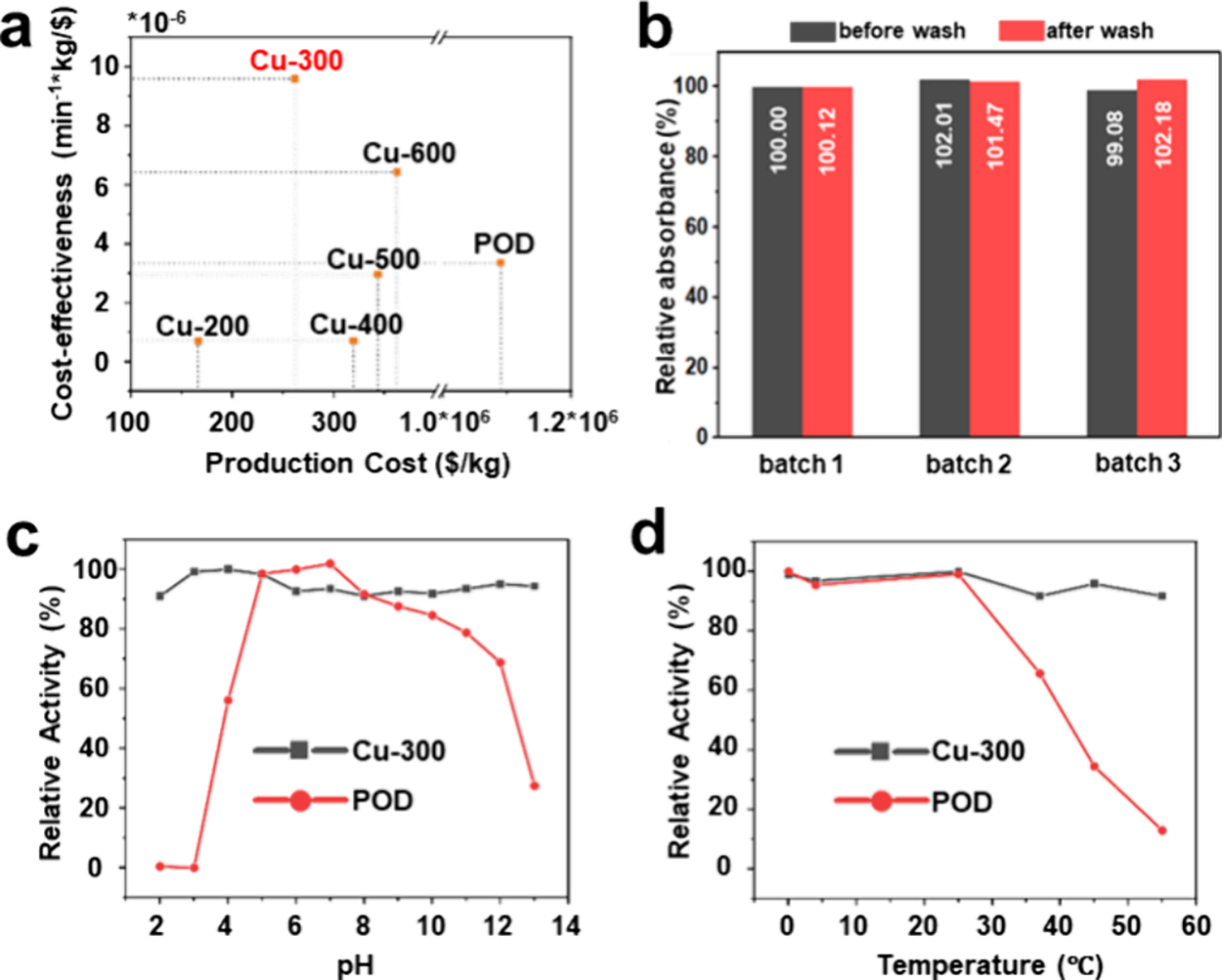
(a) Production cost and cost-effectiveness analysis of the Cu-*X* samples and POD enzymes. (b) POD-like activity of Cu-300 across different batches before and after washing. The activity of the first batch (without wash) was set as 100% for comparison. (c) pH and (d) temperature stability of Cu-300 and POD enzymes.

In addition to cost-effectiveness, long-term stability is another crucial factor for practical applications. All Cu-*X* samples were transferred to glass vials in air after their synthesis and stored under ambient conditions. The Cu-300 sample, prepared approximately 5 months ago (denoted as Cu-300–5m), was recharacterized using XRD, IR, and XPS, along with an assessment of its POD-like activity. As shown in Figure S10, negligible change can be observed in these techniques compared to Cu-300, with a similar Cu­(I)/Cu­(II) ratio, resulting in comparable POD-like activity. This result indicates that our sample is stable under ambient conditions, without the need for specialized storage. The pH and thermal stabilities of Cu-300 were further compared to those of POD enzymes. Both were pretreated in aqueous solutions at varying pH values and temperatures before assessing their ability to oxidize TMB with H_2_O_2_. As shown in Figure 4c, POD enzymes retain their catalytic ability within a pH range of 5 to 7, with activity decreasing outside this range and being significantly suppressed at pH 3 and 13. They remain fully active only at temperatures below 25 °C and experience a notable decline in activity when exposed to higher temperatures (Figure 4d). The pH and thermal instability of POD enzymes can be attributed to denaturation of their protein structures. In contrast, the POD-like activity of Cu-300 remained stable under all of the testing conditions. Its inorganic nature should further allow it to catalyze reactions at rates that theoretically double with every 10 °C increase in the temperature. The recyclability of Cu-300 was evaluated over 5 consecutive cycles (Figure S11). While activity decreased from 93% in the second run to 89% in the third, it stabilized at 83% in the subsequent runs. The initial decline may thus be attributed to the loss of some smaller particles in the sample during the recycling process.

### 3.4 Biomolecule Sensing

To emphasize the role of Cu­(I) (cf. Cu­(II)) in the POD-like activity and its performance in relevant applications, Cu-300 and Cu-400, which have contrasting Cu­(I)/Cu­(II) ratios, were further examined for the detection of GSH and glucose, as often demonstrated in the literature.^3–5^ Since GSH reduces TMB_ox_ back to colorless TMB with itself being oxidized to GSH disulfide (GSSG) (Figure 5a), the POD-like activity of both samples should reflect their sensitivity in GSH detection. As shown in Figure 5b, the characteristic absorption of TMB_ox_ at 652 nm significantly decreases in the presence of 50 μM GSH for Cu-300, whereas Cu-400 exhibits only one-fifth of this change. Consequently, Cu-300 demonstrated approximately five times greater sensitivity to GSH (Figure 5c), with an extended linear detection range from 1 to 70 μM (in contrast to 1–50 μM for Cu-400) and a lower LOD of 3.04 μM (cf. 6.43 μM for Cu-400) (Table S6). The selectivity of this sample was further evaluated against various interfering substances, displaying high selectivity for GSH (Figure 5d). To further assess the practical application of Cu-300, we performed a spiking study by adding known quantities of GSH to commercial serum samples, including human serum, horse serum, and FBS. As detailed in Table S7, this sample demonstrated nearly 100% recovery rates for GSH detection.

**Figure 5:**
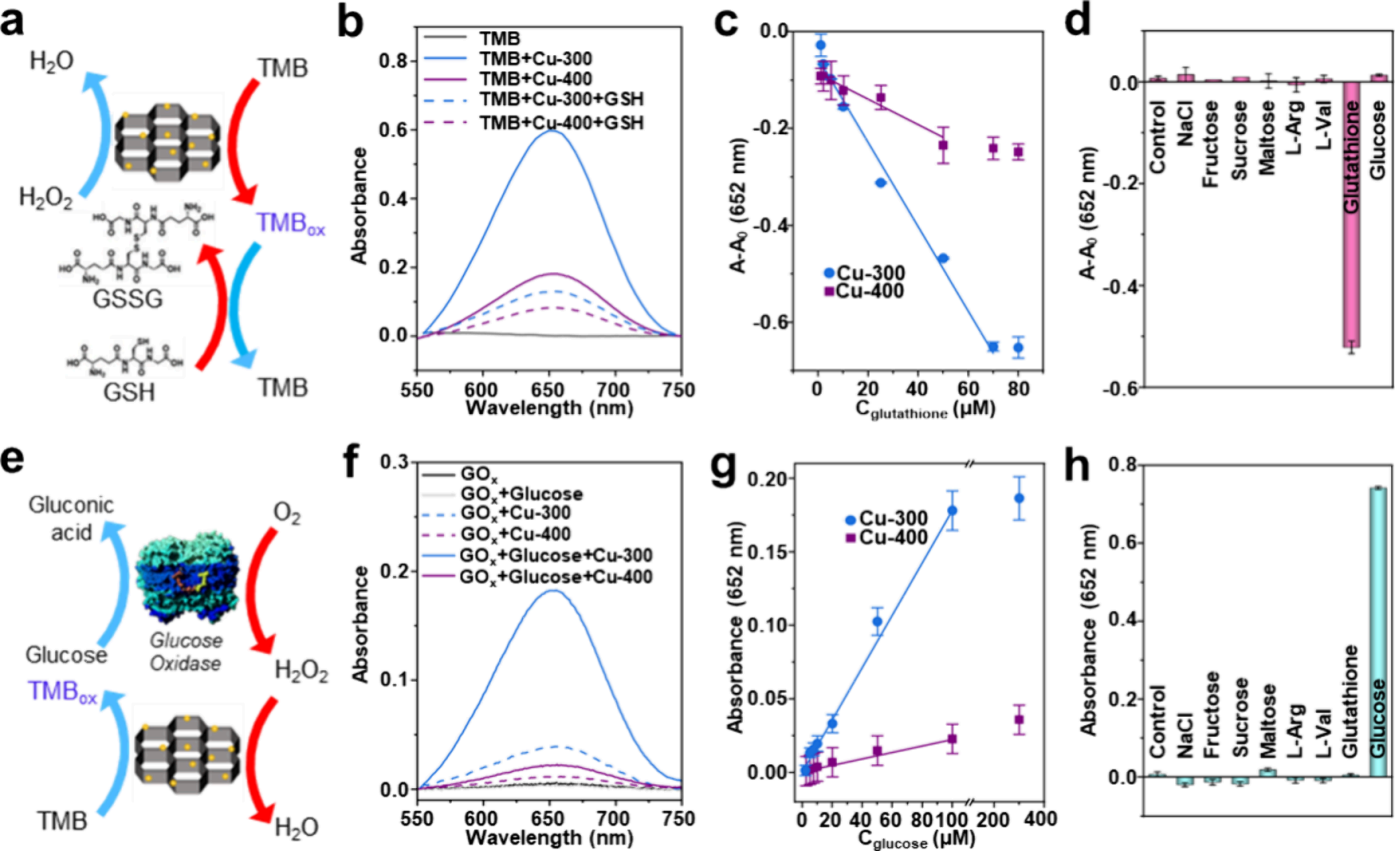
(a) Schematic illustration of GSH detection using POD nanozymes. (b) UV–vis spectra showing TMB_ox_ reduction in the presence of GSH for Cu-300 and Cu-400. (c) Absorbance change at 652 nm as a function of the GSH concentration for Cu-300 and Cu-400. (d) Selectivity test for Cu-300 in GSH detection. (e) Schematic illustration of enzyme reactions for glucose detection. (f) UV–vis spectra of glucose oxidation for Cu-300 and Cu-400. (g) Absorbance change at 652 nm as a function of glucose concentration for Cu-300 and Cu-400. (h) Selectivity test for Cu-300 in glucose detection.

In addition to GSH sensing, we also compared the performance of Cu-300 and Cu-400 as POD mimetics when combined with glucose oxidase (GO_*x*_) for glucose detection. As illustrated in Figure 5e, glucose is initially oxidized by GO_*x*_ in the presence of O_2_ to produce H_2_O_2_. This H_2_O_2_ is then reduced by the POD mimetics, resulting in the formation of TMB_ox_, which exhibits a color change that correlates with the glucose concentration. Figure 5f shows a significant increase in the absorption peak of TMB_ox_ at 652 nm for Cu-300 in the presence of 100 μM glucose, while the change is minimal for Cu-400. The response of these two samples across a range of glucose concentrations is further examined in Figure 5g, revealing that Cu-300 consistently outperforms Cu-400. Although both samples exhibit a similar linear range of 1–100 μM (Table S6), Cu-300 demonstrates over eight times greater glucose sensitivity (from the slope) and a much lower LOD of 2.57 μM (cf. 7.96 μM for Cu-400). This sample in the coupled reactions also demonstrates a high selectivity for glucose among various interferences in the solutions (Figure 5h). We further assessed the potential of substituting POD enzymes with Cu-300 in the enzymatic assay based on glucose levels from various serum samples (Figure S12). The results showed that Cu-300 can achieve ∼100% recoveries (Table S8), matching the results obtained with a commercial glucose monitoring device, thus confirming the technical feasibility of this substitution.

## 4 Conclusions

In summary, we present a facile method for preparing Cu/Cu_2_O core/shell NPs that are densely integrated into a porous carbon-based framework by calcining a mixture of Cu­(NO_3_)_2_ and PVP at elevated temperatures in nitrogen. Notably, Cu/Cu_2_O NPs were synthesized to sizes as small as approximately 15 nm at temperatures below 600 °C, with those produced at 300 °C exhibiting the highest surface Cu­(I) and corresponding POD-like activity. The free-standing porous host enables a high loading of NPs per reaction volume, which is believed to facilitate the reaction. This synthetic approach eliminates the need for purification, enabling the production of Cu-*X* samples at a cost of less than $0.4 per gram, in stark contrast to POD enzymes, which can exceed $1000 per gram. The effectiveness of the optimized sample, Cu-300, was additionally evaluated for its capability to detect GSH and glucose. This work is believed to inform the future design and synthesis of scalable, cost-effective POD nanozymes for real-world applications.

## Supplementary Material



## Notes

Experimental details including the characterization of Cu-X samples (elemental mapping, size distribution of NPs, HRTEM, XPS, infrared results); evaluation of their performance in GSH and glucose detection (PDF)
