# Supplementary material for: Facile Gram-Scale Production of Cu/Cu2O Core/Shell Nanoparticles Densely Embedded in a Porous Carbon Framework for Cost-Effective Peroxidase Mimicking
Source: ACS Appl Mater Interfaces. 2025 Jun 19;17(26):37577–85. doi: 10.1021/acsami.5c05766 (PMC12755194; doi:10.1021/acsami.5c05766)
Supplement: Supplementary file 1 [file am5c05766_si_001.pdf]

Supplementary Information

Facile Gram-Scale Production of Cu/Cu_2O Core/Shell Nanoparticles Densely Embedded in a Porous Carbon Framework for Cost-Effective Peroxidase Mimicking

Yuzhen Cai,[1#] Zhanping Xiao,[1,2#] Tianqi Cheng,[1#] Bo Yuan,[1] Yifan Cui,[1,2] Jian Lin Chen,[3] Yufei Zhao,[4] Pi-Tai Chou,[2*] and Yung-Kang Peng[1*]

[1]Department of Chemistry, City University of Hong Kong, Hong Kong 999077, Hong Kong SAR.

[2]Department of Chemistry, National Taiwan University, Taipei 106319, Taiwan.

[3]Department of Applied Science, School of Science and Technology, Hong Kong Metropolitan University, Hong Kong 999077, Hong Kong SAR.

[4]State Key Laboratory of Chemical Resource Engineering, Beijing University of Chemical Technology, Beijing 100029, China.

[#]These authors contributed equally to this work.

*Correspondence: chop@ntu.edu.tw; ykpeng@cityu.edu.hk

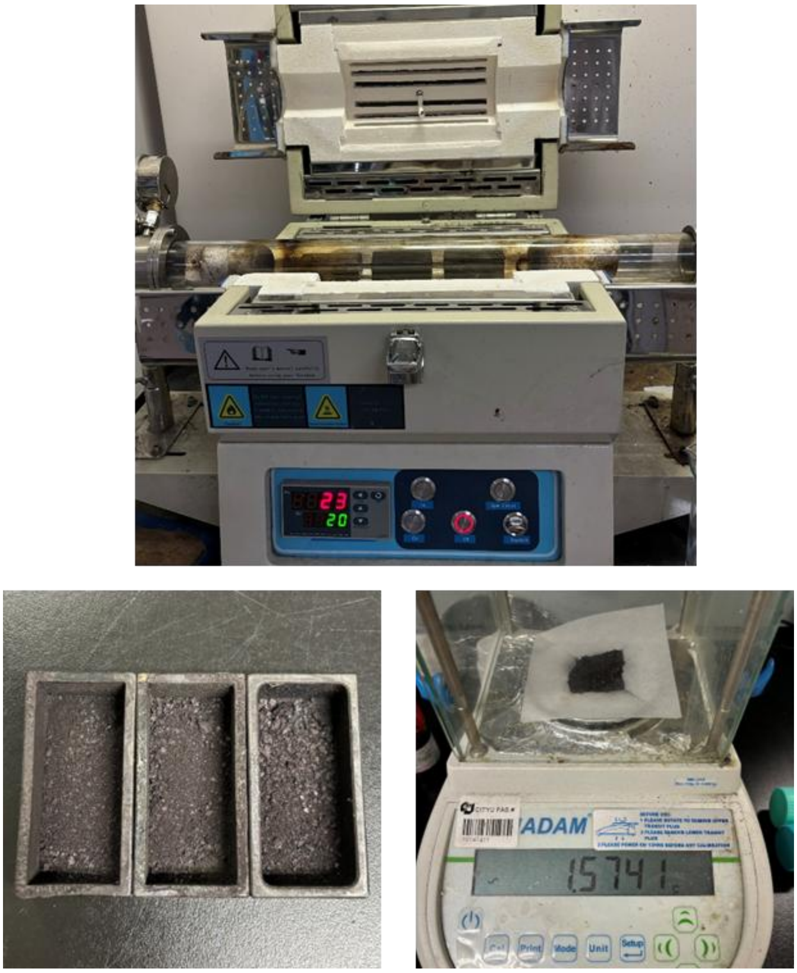

Figure S1. Images of the furnace used in our laboratory, which the tube has an inner diameter of 4.4 cm and a length of 60 cm, along with the products obtained in crucibles at 300 °C and their total weight.

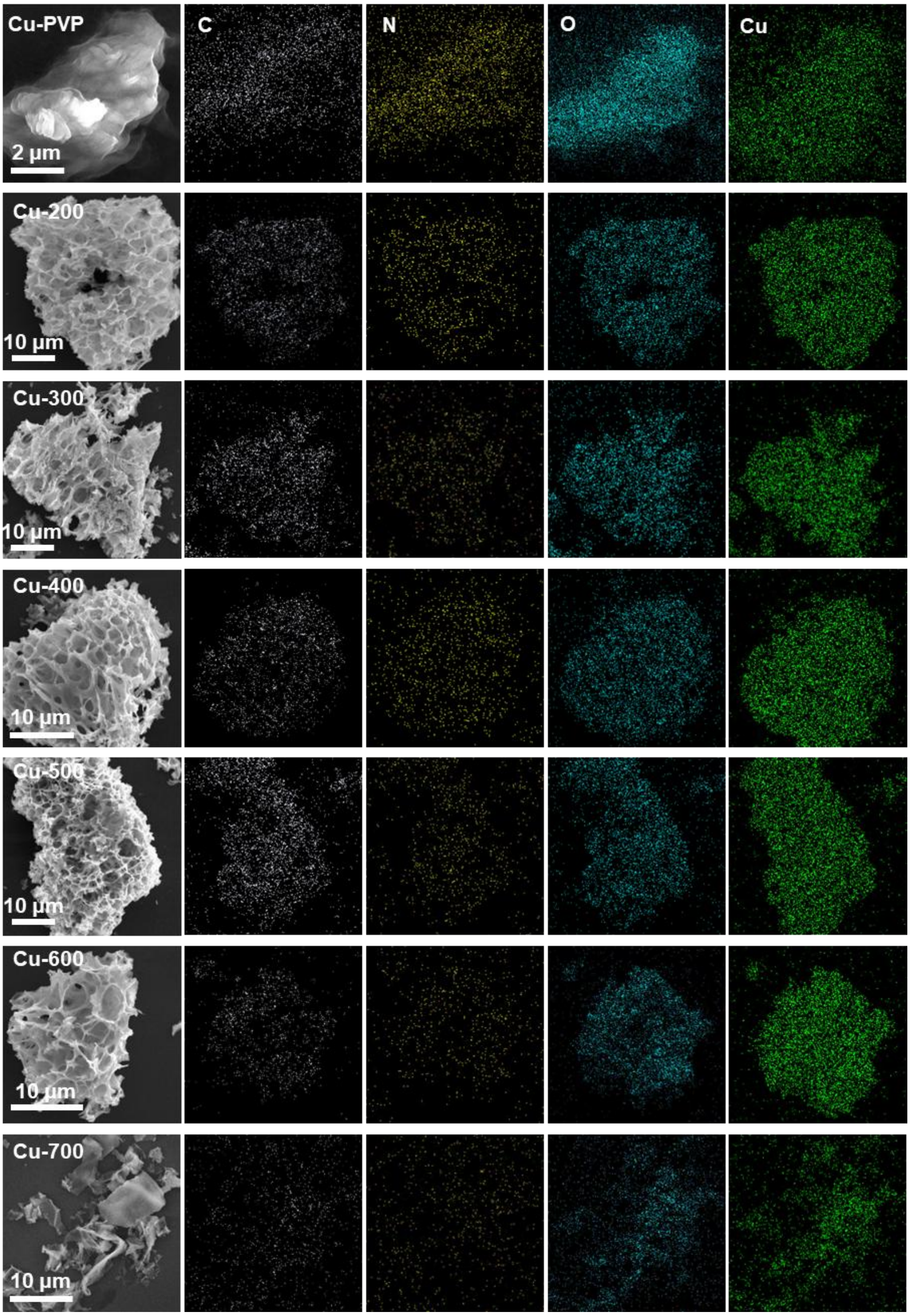

Figure S2. SEM elemental mapping of the samples.

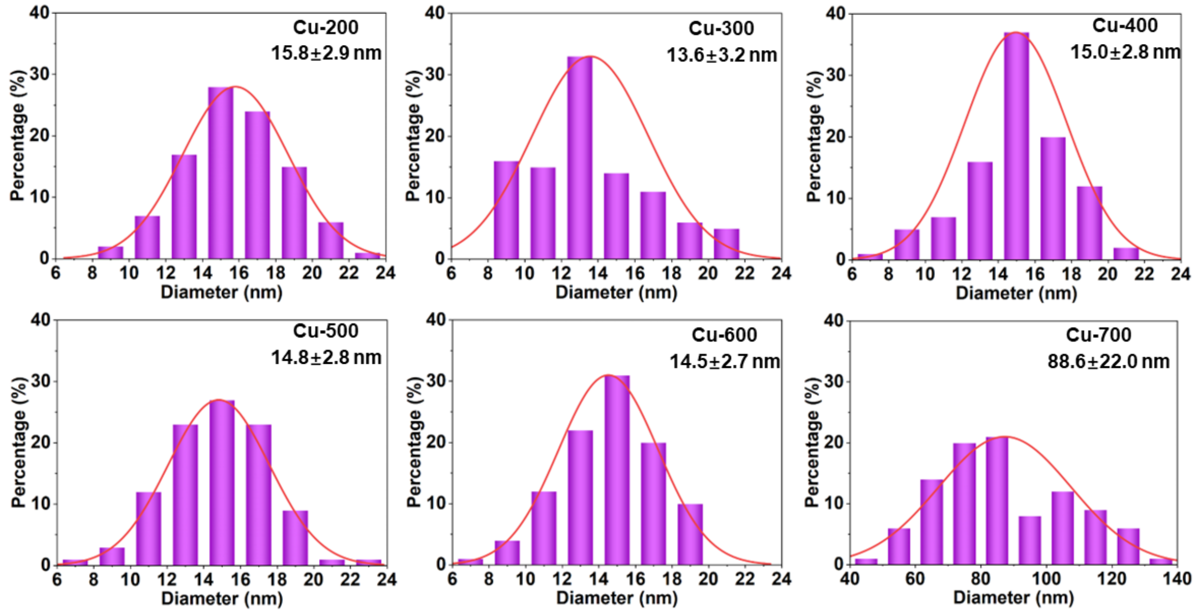

Figure S3. Size distribution of particles in the samples (100 particles counted for each sample).

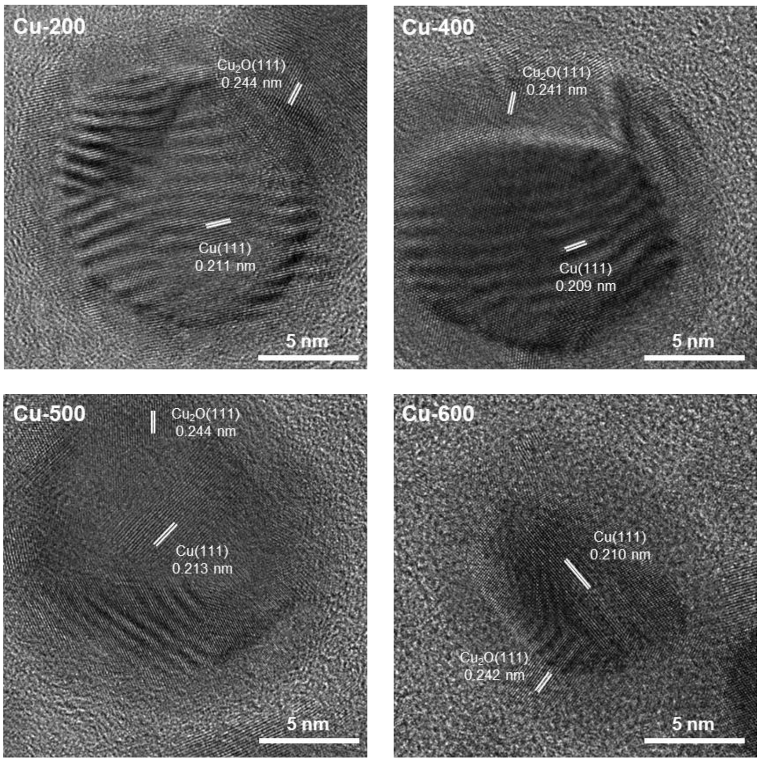

Figure S4. HRTEM images of Cu/Cu_2O NPs in the Cu-X samples.

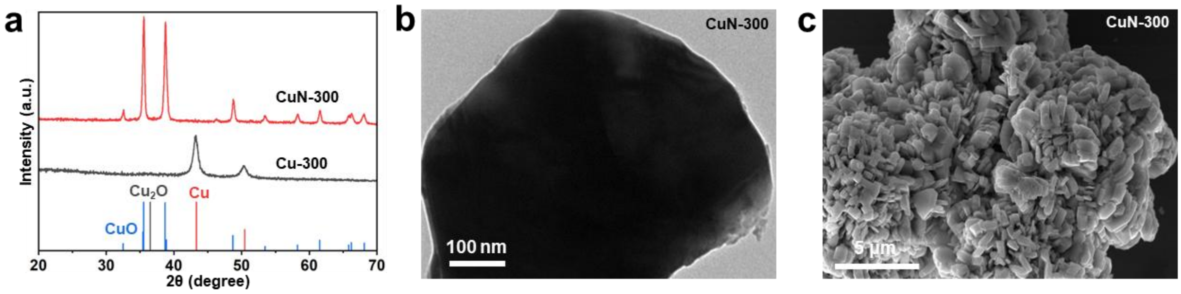

Figure S5. (a) XRD, (b) TEM, and (c) SEM results of the sample prepared by calcinating $Cu(NO_3)_2$ alone at 300 °C in N_2. The resulting sample (denoted as CuN-300) was confirmed to be Cu(II)O by XRD, in contrast to Cu(0) in Cu-300. The sharp XRD peak indicates that the particles are significantly large, a finding further supported by the TEM and SEM images.

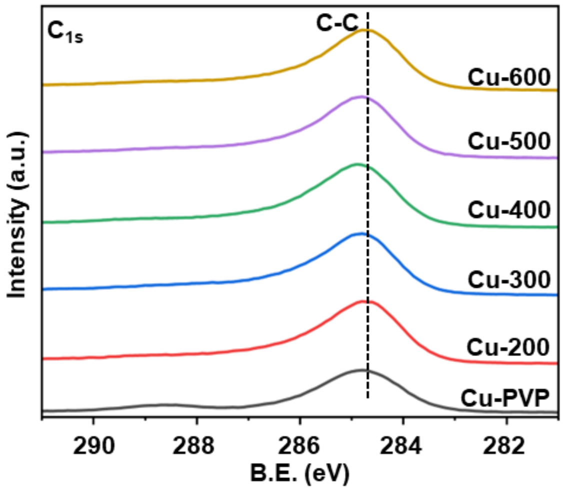

Figure S6. XPS C_{1s} spectra of the samples.

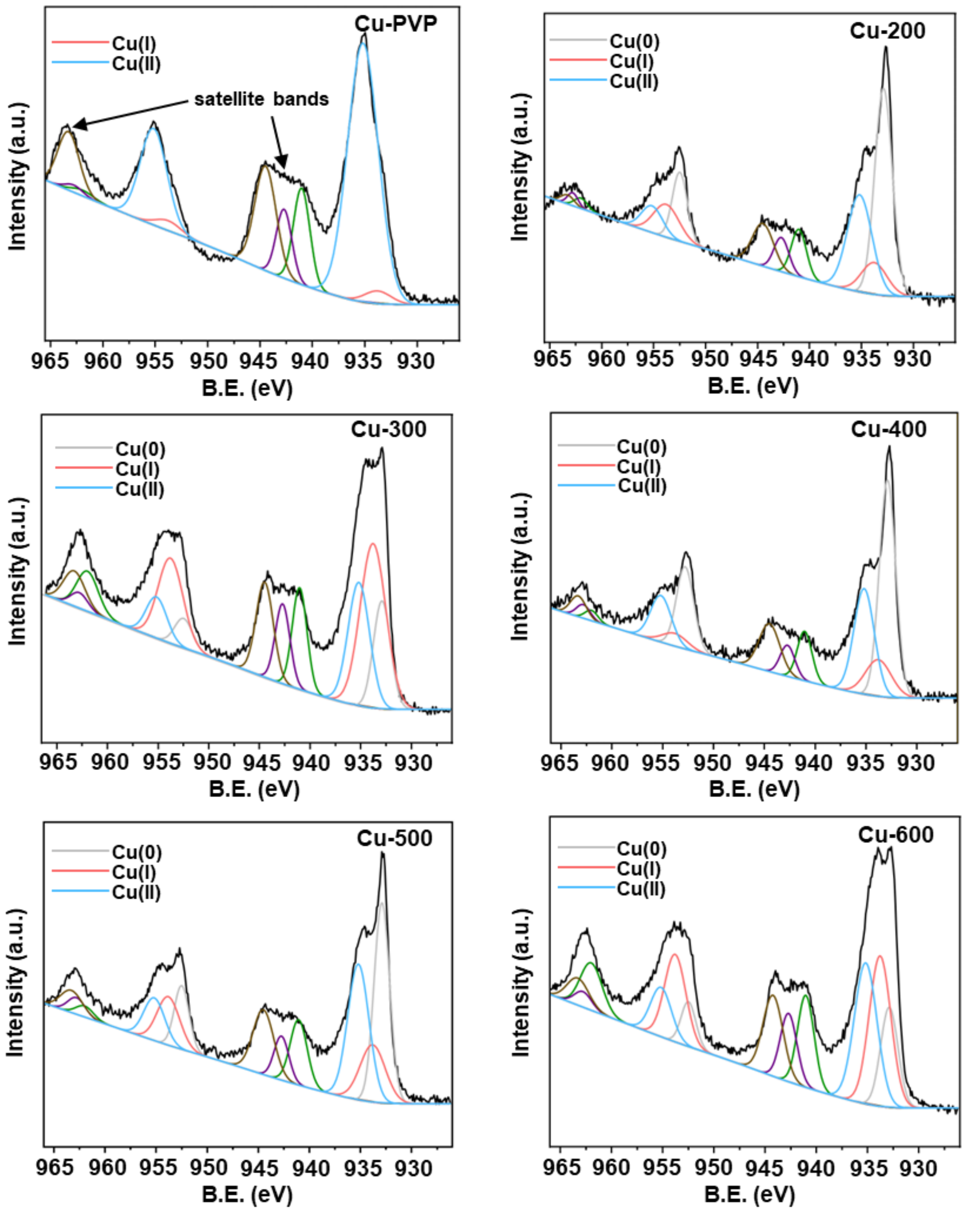

Figure S7. Deconvoluted XPS Cu_{2p} spectra of the samples.

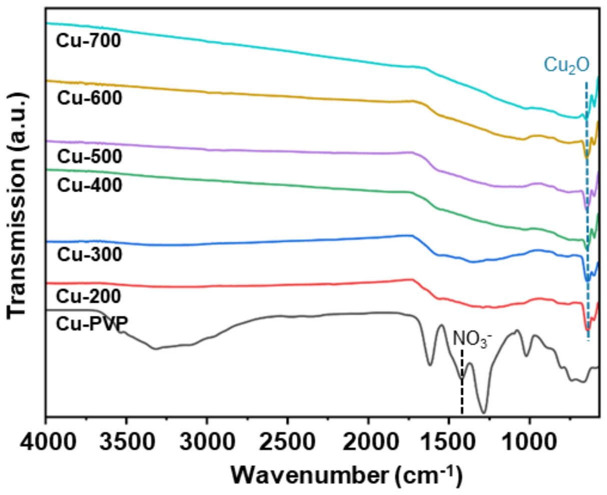

Figure S8. Infrared (IR) spectra of the samples.

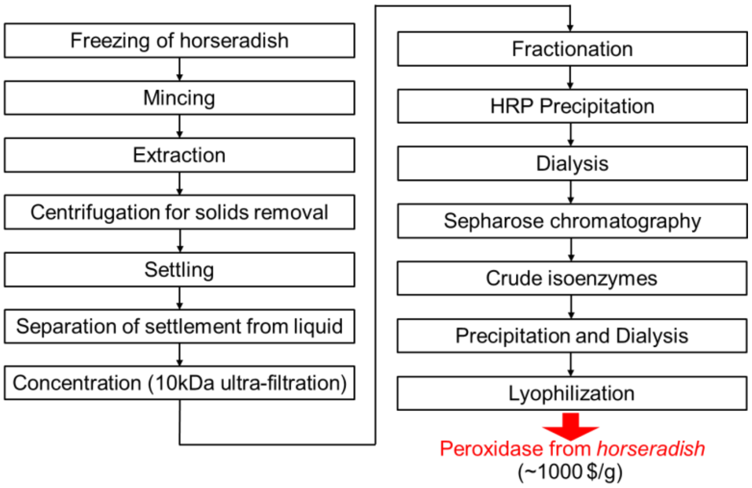

Figure S9. Schematic illustration of production processes for POD enzymes derived from horseradish.

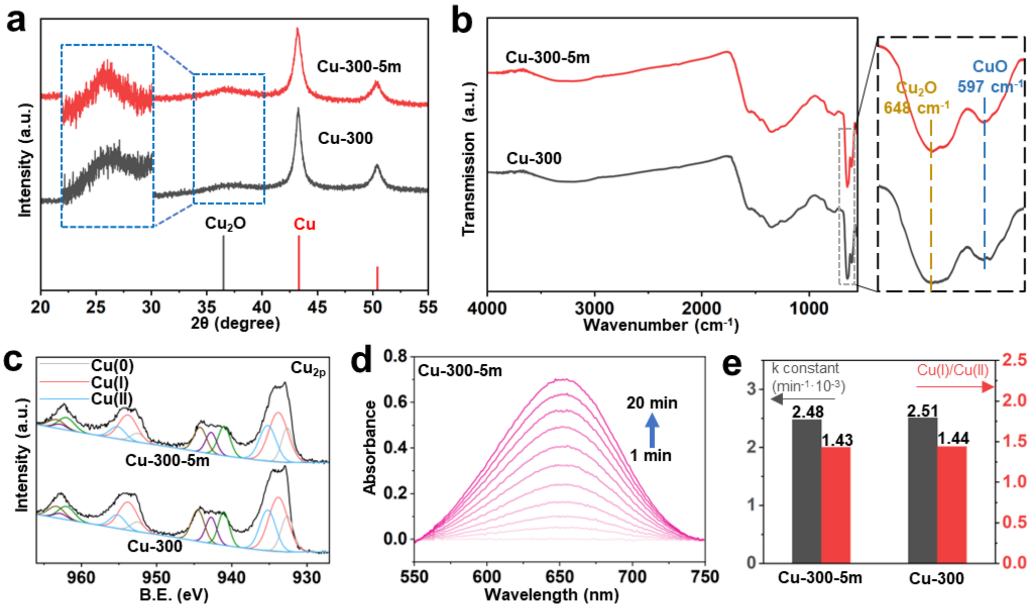

Figure S10. (a) XRD, (b) IR, and (c) Cu_{2p} XPS results for Cu-300-5m, along with (d) its POD-like activity and (e) correlation with Cu(I)/Cu(II) ratios (Cu-300 was included for comparison).

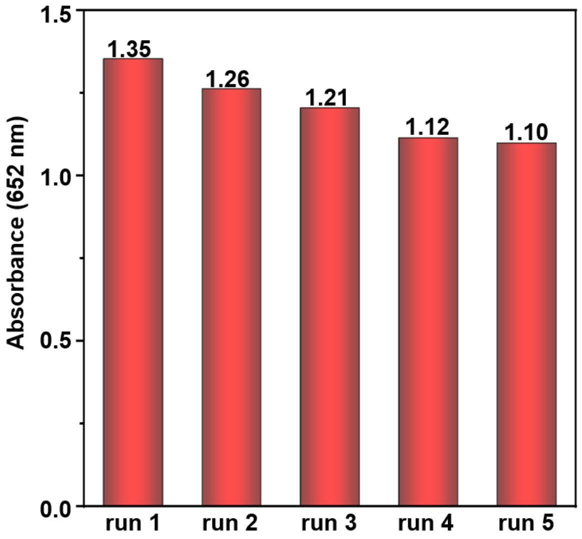

Figure S11. The activity of the Cu-300 samples over five consecutive cycles.

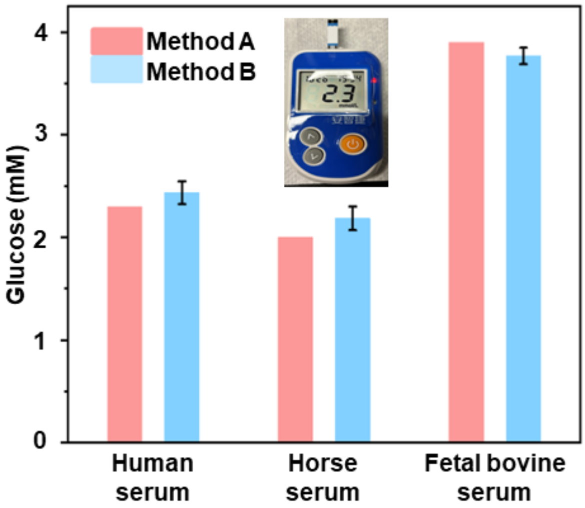

Figure S12. Comparison of the obtained glucose level in serum samples by a commercial glucose assay kit (method A) and using Cu-300 as POD mimetics (method B). Note that all error bars in this figure show the standard deviations of three replicates.

Table S1. Comparison of the catalytic performance of different POD nanozymes.

Samples	Size (nm)	k_{cat} (s^{-1})	Particle surface area (nm^2)	Normalized k_{cat} (s^{-1} nm^{-2})
V_2O_5	100 width*500 length	$2.5*10^3$	$1.73*10^5$	$1.44*10^{-2}$
Fe_2O_3	30 diameter	$1.36*10^{-1}$	$2.83*10^3$	$4.81*10^{-5}$
Fe_3O_4	19 diameter	3.03	$1.13*10^3$	$2.68*10^{-3}$
Co_3O_4	20 edge length	$1.83*10^2$	$2.4*10^3$	$7.62*10^{-2}$
NiO	32 width*65 length	$1.38*10^2$	$8.14*10^3$	$1.69*10^{-2}$
CuO	100 diameter	$7.20*10^2$	$3.14*10^4$	$2.29*10^{-2}$
Cu_2O	600 diameter	$8.71*10^5$	$9.46*10^5$	$9.20*10^{-1}$
Ru	10 diameter	$1.3*10^4$	$3.14*10^1$	$4.1*10^2$
Pd	18 edge length	$6.9*10^4$	$1.97*10^3$	$3.5*10^1$
Pt	44 edge length	$6.0*10^6$	$1.15*10^4$	$5.2*10^2$
Ir	1.5 diameter	$5.6*10^1$	7.07	7.9
Pd-Ru	20 edge length	$4.8*10^5$	$2.4*10^3$	$2.0*10^2$
Pd-Pt	42.3 edge length	$9.4*10^6$	$1.07*10^4$	$8.8*10^2$
Pd-Ir	19.2 edge length	$1.9*10^6$	$2.21*10^3$	$8.6*10^2$
Fe-Pt	1.87 diameter	$7.6*10^3$	10.98	$6.9*10^2$

Table S2. Crystallite size of Cu-X samples calculated by the Scherrer equation.

Sample	Shell Cu_2O		Core Cu	
	FWHM (2θ)	Crystallit size (nm)	FWHM (2θ)	Crystallit size (nm)
Cu-200	4.07	1.02	1.22	6.94
Cu-300	3.58	1.16	1.06	7.99
Cu-400	2.33	1.78	0.81	10.48
Cu-500	3.06	1.36	0.87	9.73
Cu-600	3.03	1.37	0.78	10.87

Table S3. Yields of products for one batch. The conversion efficiencies from raw materials to products were calculated based on the mass of residue samples after calcination, as determined by thermogravimetric analysis (TGA) **(Figure 2e)**.

Quantities of raw materials (g)	Samples	Conversion efficiencies (%)	Quantities of products (g)
0.50 g PVP and 0.75 g $Cu(NO_3)_2$	Cu-200	65.83	0.82
	Cu-300	41.65	0.52
	Cu-400	34.06	0.43
	Cu-500	31.71	0.40
	Cu-600	30.11	0.38

Table S4. The production cost and cost-effectiveness analysis of the samples, POD were conducted by considering the expenses associated with raw materials. Raw materials expenses include $Cu(NO_3)_2$ (0.12 \$/g) and PVP (0.09 \$/g) for producing 1 gram of samples. The additional costs associated with flowing N_2 and electricity used during calcination were not included in this analysis. The cost-effectiveness of each sample was determined by normalizing their rate constant (k) against the production cost.

Samples	Production cost (\$/g)	k constant ($*10^{-3}$ min^{-1})	Cost-effectiveness ($*10^{-3}$ $min^{-1}*g/\$$)
Cu-200	0.16	0.12	0.75
Cu-300	0.26	2.51	9.65
Cu-400	0.32	0.07	0.22
Cu-500	0.34	1.02	3.00
Cu-600	0.36	2.33	6.47
POD	1090.00	3660.56	3.36

Table S5. The Cu concentration of Cu-300 prepared in three different batches with/without purification measured by ICP-MS.

Sample	batch 1	batch 2	batch 3
Conc. of Cu in supernatant (mg/mL)	7.6	7.2	6.9
Conc. of Cu in sample dispersion (mg/mL)[a]	475.2	482.1	477.6
Percentage (%)	1.6	1.5	1.4

[a]The concentration of catalysts in reaction solution (1 mg/mL).

Table S6. The fitting result of targets detection.

Samples	Target	Slope (10^{-3})	SD (10^{-3})[a]	R-square	Linear range (μM)	LOD (μM)[b]
Cu-300	Glutathione	8.69	8.81	0.99	1-70	3.04
	Glucose	1.88	1.61	0.98	1-100	2.57
Cu-400	Glutathione	2.63	5.64	0.95	1-50	6.43
	Glucose	0.23	0.61	0.99	1-100	7.96

[a]SD: standard deviation. [b]LOD: limit of detection.

Table S7. Results of GSH level in serum samples determined by Cu-300.

Samples	Found in serum (μM±SD)	Spiking concentration (μM)	Concentration obtained (μM±SD)	RSD (%)[a]	Recovery (%)[b]
Human serum	6.6	10	16.1±0.6	3.7	96.8
		20	25.5±1.0	4.1	96.0
		30	35.8±0.8	2.4	97.9
Horse serum	6.2	10	15.8±0.7	4.5	97.3
		20	25.6±0.6	2.4	97.6
		30	35.2±0.5	1.5	97.1
Fetal bovine serum	9.1	10	18.5±0.3	1.8	96.7
		20	28.1±0.3	1.1	96.4
		30	38.4±0.4	1.1	98.3

[a]The relative standard deviation (RSD) was obtained by dividing the standard deviation by the corresponding average value. [b]Recovery was calculated by dividing the averaged concentration obtained by the spiking concentration.

Table S8. Results of glucose level in serum samples determined by a commercial glucose assay kit (method A) and using Cu-300 as POD mimetics (method B).

Samples	Method A (mM)	Method B (mM)	RSD (%)[a]	Recovery (%)[b]
Human serum	2.3	2.4±0.1	4.5	104.3
Horse serum	2.0	2.2±0.1	5.3	110.0
Fetal bovine serum	3.9	3.8±0.1	2.1	97.4

[a]The RSD was obtained by dividing the standard deviation by the corresponding average value. [b]Recovery was calculated by dividing the averaged concentration obtained in method B by method A.
